# Clonal distribution of bone sialoprotein-binding protein gene among *Staphylococcus aureus* isolates associated with bloodstream infections

**DOI:** 10.1007/s12223-014-0321-7

**Published:** 2014-05-15

**Authors:** Katarzyna Wiśniewska, Anna Piórkowska, Joanna Kasprzyk, Marek Bronk, Krystyna Świeć

**Affiliations:** 1 Department of Medical Microbiology, Medical University of Gdańsk, Do Studzienki 38, 80-227 Gdańsk, Poland; 2 Laboratory of Clinical Microbiology, University Clinical Centre in Gdańsk, Dębinki 7, 80-952 Gdańsk, Poland

## Abstract

*Staphylococcus aureus* is a leading cause of bloodstream infections (BSI) and diseases that may be caused by hematogenous spread. The staphylococcal adhesin, for which the association with the infections emerging as a complication of septicemia has been well documented, is a bone sialoprotein-binding protein (Bbp). The aim of the study was to assess the prevalence of a *bbp* gene in *S. aureus* bloodstream isolates associated with BSI and to investigate to what degree the distribution of this gene is linked to the clonality of the population. *Spa* typing, used in order to explore the genetic population structure of the isolates, yielded 29 types. Six *spa* clusters and seven singletons were identified. The most frequent was *spa* clonal complex CC021 associated with MLST CC30 (38 %). The *bbp* gene was found in 47 % of isolates. Almost all isolates (95 %) clustered in *spa* clonal complex CC021 were positive for this gene. All isolates carrying the *bbp* gene were sensitive to methicillin, and if clustered in the *spa* CC021, belonged to *agr* group III. Our study shows that Bbp is not strictly associated with BSI. However, one may conclude that for clonally related *S. aureus* strains most commonly causing BSI, the risk of Bbp-mediated complications of septicemia is expected to be higher than for other strains.

## Introduction

*Staphylococcus aureus* is a leading cause of bloodstream infections (BSI) (Grundmann et al. 2010). Hospital BSIs caused by this bacterium are associated with significant morbidity and mortality, particularly in populations at high risk of infection. An early and critical step in establishing BSI process is bacterial adhesion and colonization of host tissues (Naber 2009). In *S. aureus*, surface proteins interacting with host molecules were named *Microbial Surface Components Recognizing Adhesive Matrix Molecules* (MSCRAMMs) (Patti and Höök 1994 *).* The relationship between the tissue tropism and the expression of particular MSCRAMMs is still insufficiently cleared but the adhesin, for which the association with the infections emerging as a complication of septicemia has been well documented, is a bone sialoprotein-binding protein (Bbp) (Cunningham et al. 1996; Rydén et al. 1987, 1989). Moreover, there is evidence that the role of Bbp can be broader than that of a simple tissue tropism (Persson et al. 2009; Vazquez et al. 2011).

The Bbp interacts with bone sialoprotein (BSP), an essential component of bone and dentine extracellular matrix (Yacoub et al. 1994). It is possible that Bbp may function in two capacities: as an important factor in the colonization of bone tissue and as a contributing factor in *S. aureus* hematologic diseases (Berendt and Byren 2004). In an in vivo animal model, BSP binding capacity was found in all staphylococci-producing septic arthritis (Bremell et al. 1991). Moreover, the serum from patients suffering from bone and joint infection contained antibodies that reacted with the fusion protein of the BSP-binding protein, indicating that the protein is expressed during an infection and is immunogenic (Persson et al. 2009). The study of Vazquez et al. (2011) showed that human fibrinogen is also a ligand for Bbp which can manipulate the biology of the fibrinogen in the host (Vazquez et al. 2011). Thus, a specificity of Bbp for a factor other than BSP cannot be excluded.

The diseases preferentially associated with Bbp are osteomyelitis and septic arthritis (Bremell et al. 1991; Rydén et al. 1987, 1997). The presence of *bbp* gene encoding for Bbp in *S. aureus*-caused orthopedic implant infections has been described (Campoccia et al. 2009). Moreover, co-existence of *bbp* and collagen-binding protein (*cna*) gene in such isolates suggests that these two adhesins may act together in the implant infections (Campoccia et al. 2009). The combination of the *bbp* and *cna* genes was also found in the pandemic type of community-acquired methicillin-resistant *S. aureus* (CA-MRSA) (Arnold et al. 2006). According to evidence, the expression of *S. aureus bbp* gene may correlate with genes for methicillin-resistance and Panton-Valentine leukocidin (PVL) (Otsuka et al. 2006; Witte et al. 2005). The association between the accessory gene regulator (*agr*) system and *bbp* presence in *S. aureus* has also been described (Montanaro et al. 2010).

The species *S. aureus* has a highly clonal population structure (Feil et al. 2003) but the frequency of Bbp in *S. aureus* isolates causing bacteremia has not been well documented. Moreover, the diseases associated with Bbp may be a complication of septicemia as the staphylococci reach a bone or joint region via the bloodstream. For this reason, the aim of the present study was to assess the prevalence of the *bbp* gene in *S. aureus* isolates associated with BSI and to investigate to what degree the distribution of this gene is linked to the clonality of the population.

## Materials and methods

### Bacterial isolates

The study was performed on 53 *S. aureus* bloodstream isolates obtained from patients of 19 departments of the University Clinical Center (UCC) in Gdańsk, including the following: cardiology (12); nefrology, transplantology, and internal medicine—NTIM (6); hematology (4); internal medicine, endocrinology, and homeostasis disturbances—IMEHD (4); gastrohepatology (3); hypertension and diabetology (3); adult neurology (3); intensive care unit—ICU (3); oncology and radiology (2); internal medicine and intoxications—IMI (2); isolation unit (2); cardiac surgery; hypertension, internal medicine, hemodialysis, and transplant medicine—HIMHTM; venerology and allergology; rehabilitation; pediatric nefrology; pediatric oncology, hematology and endocrinology—POHE; neurological surgery (1 each); not classified (2). All the patients were considered to have true bloodstream infection defined as at least one blood culture positive for *S. aureus* during the presence of the systemic inflammatory response syndrome. The additional data about clinical recognitions and risk factors associated with BSI in individual patients were not available. The strains were isolated in the UCC laboratory from March 2008 to March 2009 (13 months) and sent to the Microbiology Department of the Medical University of Gdańsk for further analyses. The first isolate from patient was included in the study. The bacteria were cultured on sheep blood agar (Oxoid, UK) and were re-identified by API ID32 Staph system (Bio Mérieux, France). For further purposes, the isolates were subcultured on nutrient broth and stored in glycerol at −70 °C.

### Bacterial DNA isolation

Genomic DNA used as an amplification template was extracted from the bacterial cultures using Gene MATRIX Bacterial & Yeast Genomic DNA Purification Kit (EURx Ltd., Poland) according to the manufacturer’s instruction.

### Antimicrobial drug susceptibility testing

Resistance to methicillin was primary tested using disc diffusion method on Mueller Hinton agar (Oxoid, UK), with oxacillin and cefoxitin discs (Becton Dickinson, Germany) and confirmed by the detection of *mecA* gene by polymerase chain reaction (PCR) (Barski et al. 1996). The susceptibility to antimicrobial agents was determined by the disc diffusion method according to recommendations given by Hryniewicz et al. (2005) and the CLSI guidelines (2006). The antibiotics tested were the following: penicillin, erythromycin, clindamycin, ciprofloxacin, co-trimoxazole, tetracycline, gentamicin, vancomycin, teicoplanin, fusidic acid, rifampicin, linezolid, quinupristin-dalfopristin, chloramphenicol (Becton Dickinson, Germany), and mupirocin (Oxoid, UK). For all the isolates beta-lactamase production was checked by nitrocefin test (Becton Dickinson, Germany). For isolates identified as resistant to erythromycin, but susceptible to clindamycin, *D*-test was performed to detect inducible clindamycin resistance (Fiebelkorn et al. 2003). The susceptibility to vancomycin was confirmed by the minimal inhibitory concentration (MIC) using *E*-tests, as described by the manufacturer (AB Biodisc, Sweden).

### Detection of *bbp* and *cna* genes

Detection of *bbp* and *cna* genes was performed by PCR. The nucleotide sequence of the primers and thermal cycling conditions were described by Tristan et al. (2003).

### Detection of *PVL* genes

All the isolates were screened for the presence of *lukS*-PV and l*ukF*-PV genes by PCR as described by Lina et al. (1999).

### *Spa* typing and based upon repeat pattern analysis

The polymorphic X region of the protein A gene (*spa*) was amplified from the isolates by PCR according to the procedure described by Harmsen et al. (2003). *Spa* types were determined by using Ridom Staph Type software (Harmsen et al. 2003). The *spa* types were clustered into *spa* CCs (clonal complexes) using the algorithm based upon repeat pattern (BURP) (Rupptisch et al. 2006).

### Determination of MLST clonal complexes

Since *spa* typing, together with the algorithm BURP, and multilocus sequence typing (MLST) are highly concordant (Enright et al. 2000; Strommenger et al. 2008), the associated MLST CCs were allocated through the Ridom SpaServer database (www.spaserver.ridom.de).

### *Agr* typing

The a*gr* group-specific multiplex PCR was performed with the use of primers and thermal cycling conditions described previously (Shopsin et al. 2003).

### SCC*mec* typing

Typing of the staphylococcal chromosomal cassette (*SCC) mec* was done in MRSA isolates as described previously (Milheiriço et al. 2007).

## Results

The results of the current *bbp* gene research, in relation to *spa* typing and clonal complexes analysis, are provided in Table 1. *Spa*–typing yielded 29 types. BURP analysis of the *spa* types identified six clusters and seven singletons. The most frequent was *spa* CC021, associated with MLST CC30, harboring 38 % of the isolates. Within the *spa* CC021, consisted of nine types, the most common was t021 (7/20 = 35 %). The second frequent was singleton represented by 13 % of the isolates, with only one *spa* type t127 related to MLST CC1. The third one was *spa* CC085 (9 %) consisted of three types: t084, t085, t355 associated with MLST CC15. The *bbp* gene was found in 47 % of the isolates. Most of the isolates positive for *bbp* belonged to the *spa* CC021 (76 %). The *bbp* gene occurred in 19/20 isolates of this cluster (95 %). The remaining isolates carrying the *bbp* gene were included in a cluster without the primary founder of a group (t645 and t4495), associated with MLST CC121, or in *spa* CC775 related to MLST CC25 (t056 and t775). One isolate with *spa* repeat pattern uncorrelated with other types was also positive for *bbp.* No one isolate belonging to the most frequent singleton or to the *spa* CC085 carried the *bbp* gene.

**Table 1:** Occurrence of the bone sialoprotein-binding protein gene in *S. aureus* bloodstream isolates, in relation to *spa* types and clonal complexes

*Spa* complex	Number (%) of isolates	Number of *spa* types	*spa* type (number of isolates)	Number (%) of the *bbp* gene-positive isolates	AssociatedMLST CC^a^
CC021	20 (38 )	9	t021(7); t012 (3); t12362 (3); t342 (2); t10689 (1); t122 (1); t300 (1); t017(1); t1649 (1)	19 (76)	30
CC085	5 (9)	3	t084 (3); t085 (1); t335 (1)		15
CC775	4 (8)	3	t078 (2); t056 (1); t775 (1)	2 (8)	25
No founder #4	4 (8)	2	t015 (3); t050 (1)		45
No founder #5	3 (6)	2	t645 (2); t4495 (1)	3 (12)	121
No founder #6	3 (6)	3	t005 (1); t032 (1); t310 (1)		22
Singleton #1	2 (4)	1	t008 (2)		8
Singleton #2	7 (13)	1	t127 (7)		1
Singleton #3	1 (2)	1	t148 (1)		72
Singleton #4	1 (2)	1	t164 (1)		20
Singleton #5	1 (2)	1	t1508 (1)		–
Singleton #6	1 (2)	1	t003 (1)		5
Singleton #7	1 (2)	1	t9489 (1)	1 (4)	–
Total	53 (100)	29 (100)	–	25 (100)	–

^a^CC, as allocated though the Ridom SpaServer database

Table 2 shows that in the total studied population only three isolates (6 %) were resistant to methicillin. The MRSA isolates were identified as t032/*Sccmec*IV, t008/*Sccmec*IV, and t003/*Sccmec*II (shown in footnote below the table). None of the MRSA isolates were positive for *bbp*, whereas 3/7 of the *PVL*-positive isolates carried the *bbp* gene. The *cna* gene was detected in 33/53 isolates (62 %) of which 19 (58 %) also carried *bbp*. The *agr* polymorphism study revealed that the most frequent were the *agr* types III (29/53 = 55 %) and I (14/53 = 26 %). The *bbp* gene possessed 69 % of the isolates of the *agr* group III, 14 % of the isolates of the *agr* group I and all the isolates of the *agr* group IV. No isolate belonging to the *agr* group II was positive for *bbp*.

**Table 2:** Occurrence of the *bbp* gene in 53 *S. aureus* bloodstream isolates with regard to methicillin-resistance and presence of selected virulence associated determinants

Isolates	Number (%) of isolates							
	MSSA	MRSA	*PVL*+	*cna*+	*agr* group			
					I	II	III	IV
*bbp+*	25 (50)	0	3 (43)	19 (58)	2 (14)	0	20 (69)	3 (100)
*bbp*−	25 (50)	3 (100)^a^	4 (57)	14 (42)	12 (86)	7 (100)	9 (31)	0
Total	50 (100)	3 (100)	7 (100)	33 (100)	14 (100)	7 (100)	29 (100)	3(100)

*bbp* bone sialoprotein-binding protein gene, *PVL* Panton-Valentine leukocidin toxin gene, *cna* collagen-binding protein gene, *agr* accessory gene regulator

^a^t032/*Scc*mec IV; t008/*Scc*mec IV; t003/*Scc*mec II

The subpopulation of isolates positive for *bbp* was described in Table 3. According to our data, such isolates were distributed in 16 departments and were constantly present in the UCC throughout the observed period. The *bbp* positive isolates were obtained with the highest frequency from patients of the cardiology (16 %) and the nefrology, transplantology, and internal medicine department (12 %). Almost all isolates with the *bbp* gene (92 %) were resistant to penicillin. All penicillin-resistant isolates produced beta-lactamase (data not shown). Resistance to penicillin only was observed in 56 % of isolates, in terms of the *spa* CC021 and one of the clusters without the primary founder of a group. In five isolates (20 %) exhibiting penicillin resistance, additional resistance to macrolides, inducible or constitutive, was observed. Three isolates (12 %), including two belonging to the *spa* CC775/*agr* type I and one to CC021, were sensitive to all the antibiotics. Only one isolate was resistant to penicillin, macrolides, ciprofloxacin, tetracycline, and chloramphenicol. The isolates harboring the *bbp* gene belonged to *agr* group III in 84 % were positive for *cna* gene in 88 % and for *PVL* in 12 %. None of the isolates belonging to the *spa* CC021 harbored the *PVL* gene.

**Table 3:** Characteristic of the bone sialoprotein-binding protein gene positive *S. aureus* bloodstream isolates

No.	Date of isolation	Department	Antibiotic resistance	*Spa* type	*spa-*CC	*PVL*	*cna*	*agr* group
1.	8.03	Cardiology	Pe	t021	CC021	–	+	III
2.	5.05.	Cardiology	Pe E CC	t122	CC021	–	+	III
3.	9.05.	Gastrohepatology	Pe E Cc Cip Te C	t645	Nf #5	–	+	IV
4.	19.05.	Oncology and Radiology	Pe E Cc/	t021	CC021	–	+	III
5.	26.05.	Cardiac Surgery	Pe	t021	CC021	–	+	III
6.	28.05.	NTIM	Pe	t4495	Nf #5	–	+	IV
7.	5.06.	HIMHTM	Pe	t12362	CC021	–	+	III
8.	11.06.	Hematology	Pe	t10689	CC021	–	+	III
9.	2.07.	Isolation Unit	Pe	t021	CC021	–	+	III
10.	18.07	Cardiology ICU	Pe	t342	CC021	–	+	III
11.	2.10.	Gastrohepatology	Pe	t645	Nf #5	+	+	III
12.	16.10.	Oncology and Radiology	Pe E Cc	t012	CC021	–	+	III
13.	14.10.	Intensive Therapy	Pe Te	t021	CC021	–	+	III
14.	9.11	Rehabilitation	Pe Cip	t012	CC021	–	+	III
15.	10.11	NTIM	Pe	t021	CC021	–	+	III
16.	27.11	NTIM	Senistive	t017	CC021	–	+	III
17.	29.11	Adult Neurology	Pe E Te	t12362	CC021	–	+	III
18.	25.12	IMEHD	Pe E Cc/	t12362	CC021	–	+	III
19.	28.12	No data	Pe	t021	CC021	–	–	III
20.	10.01.	Hematology	Sensitive	t056	CC775	+	–	I
21.	15.01	POHE	Pe	t1649	CC021	–	+	III
22.	27.01	Cardiology ICU	Sensitive	t775	CC775	+	–	I
23.	6.02	Neurosurgery	Pe	t342	CC021	–	+	III
24.	27.02	Adult Neurology	Pe	t9489	singleton	–	+	III
25.	26.03	IMI	Pe	t012	CC021	–	+	III

*NTIM* nefrology, transplantology, and internal medicine, *HIMHTM* hypertension, internal medicine, hemodialysis, and transplant medicine, *ICU* intensive care unit, *IMEHED* internal medicine, endocrinology, and homeostasis disturbances, *POHE* pediatric oncology, hematology and endocrinology, *IMI* internal medicine and intoxications—as described in “Materials and methods” section; *Pe* penicillin, *E* erythromycin, *Cc* clindamycin, *Cip* ciprofloxacin, *Te* tetracycline, / inducible clindamycin resistance, *Nf* no founder cluster, *PVL* Panton-Valentine leukocidin toxin gene, *cna* collagen-binding protein gene, *agr* accessory gene regulator

## Discussion

In the first step of our investigations, we focused on molecular characterization of the *S. aureus* isolates associated with BSI. In order to explore the genetic population structure of the isolates, we used *spa* genotyping as a method with both a high discriminatory power and results that can be easily compared between laboratories (Strommenger et al. 2008). *Spa* typing is based on the variation of the polymorphic region within the staphylococcal protein A gene (Harmsen et al. 2003). There is a high concordance between *spa* typing and other genotyping methods, including MLST (Enright et al. 2000; Strommenger et al. 2008). Thus, we analyzed the results considering the relationship to MLST clones described in database.

Looking at the clonal structure of the isolates, our results confirm several major findings of previously published surveys (Fenner et al. 2003; Grundmann et al. 2010; Nulens et al. 2008). The most frequent in our collection was *spa* CC021 related to MLST CC30, with the main *spa* type t021. According to the data obtained by international multicenter trials, t021 belongs to the 20 most frequent *spa* types among *S. aureus* isolates causing invasive infections in Europe (Grundmann et al. 2010). We confirmed findings of a study on local population of *S. aureus* in Eastern Pomerania, Poland, where within CC30, the t021 dominated the bloodstream isolates (Holtfreter et al. 2007). But, interestingly, in that study, CC30 was significantly more common among nasal strains than among blood culture isolates. It could mean that CC30 is widely disseminated among Polish *S. aureus* strains population in general. In contrast, in other European countries, CC30 is reported as more prevalent in invasive strains than in noninvasive (Wertheim et al. 2005). For example, *spa* types corresponding with CC30 were observed in invasive MSSA in a Swiss University Hospital (Fenner et al. 2003) and in bloodstream isolates collected from a Dutch University Hospital (Nulens et al. 2008). The more recent study from Norway showed that CC30 was the most frequent clonal complex in MSSA isolates from deep surgical site infections in orthopedic patients (Aamot et al. 2012). However, there were some differences, when comparing our results to data published by others. The t002/ST-5 and t084/ST-15 were described as the first *spa* types among MSSA from invasive infections in Europe in 2010. In Polish laboratories participating in that study, the most prevalent among MSSA were t127/ST-1, t084/ST-15, and t015/ST-45 (Grundmann et al. 2010). In our collection, t127 was observed as frequently as t021 but with no correlation to other *spa* types and t002 was not found at all.

The main conception of our study was to assess the prevalence of the *bbp* gene, in relation to the clonality of the population described above. The results could mean that Bbp is not strongly associated with BSI, as the gene for this factor was found in almost half of the isolates. It may be not surprising, since our strain collection was not restricted to patients with selected risk factors for a particular location of infection, as it has been described in the literature (Aamot et al. 2012; Luedicke et al. 2010; Rydén et al. 1989). For example, in the Aamot et al. study (2010) on MSSA isolates from deep surgical site infections in orthopedic patients, 95 % of the isolates from invasive diseases were positive for *bbp*. Moreover, our investigations were not restricted to the departments where, according to evidence, the *bbp-*positive strains among *S. aureus* isolates were supposed to occur with the highest frequency (Aamot et al. 2012). But the findings of previously published investigations on *S. aureus* strains associated with blood infections were not consistent. For example, in the study of Tristan et al. (2003), the *bbp* gene was strictly associated with *S. aureus* strains involved in human hematogenous infections. By contrast, in the Hogevik et al. report (1998), no correlation was found between the potential bacterial virulence factors, including Bbp and infective endocarditis. What comes to our notice is that almost all *bbp* positive isolates were clustered to the *spa* CC021, corresponding to MLST CC30—the most widely disseminated clonal complex among the *S. aureus* bloodstream isolates from the UCC. It is interesting in view of the hypothesis that CC30 causes systemic infections only under very accommodating conditions (Holtfreter et al. 2007).

The production of toxins and surface proteins in *S. aureus* is globally regulated by the *agr* system. Four allelic groups of *agr* have been recognized in *S. aureus* (Novick et al. 1993). Some epidemiological findings suggest that the particular *agr* group can correspond to the genetic background in this bacterium and may be associated with virulence profile suitable to clinically specific infections (Montanaro et al. 2010). In our study, the most frequently observed *agr III* group was generally associated with the presence of the *bbp* gene, whereas the second one *agr* I was rather negative. Additionally, a few isolates of *agr* IV showed full association with the *bbp* gene, in contrast to *agr* II, fully negative for this gene. The similar observation was done by Montanaro et al. (2010) in *S. aureus* isolates from orthopedic implants infections. They showed that *agr* III differed clearly from *agr* I and II, exhibiting high prevalence of *bbp*, whereas *agr* I and II generally did not harbor this gene. Moreover, the same authors described *agr* II in such isolates as to be frequent in epidemic clones.

The association between the presence of *bbp* and *cna* genes, similar to that found in *S. aureus* causing orthopedic implant infections (Campoccia et al. 2009) was not observed in our bloodstream isolates. The frequencies of the *cna* gene in *bbp*-negative and *bbp*-positive isolates were similar. However, most of the isolates carrying the *bbp* gene were positive for *cna*. Thus, the conclusion that these two adhesins act together in BSI may not be excluded. It is clear that the interactions of *S. aureus* with host cells are complex.

The lack of the *bbp* gene in any MRSA strains may be explained by relatively low prevalence of MRSA among bloodstream isolates at the UCC in the study period. Our earlier investigations, performed on MRSA population in this hospital, revealed the predominance of *spa* CC010 and *spa* types related to CA-MRSA (Wiśniewska et al. 2012). The present study confirmed that in the bloodstream collection isolates two *spa* types associated with CA-MRSA: t008/SCC*mec*IV and t0032/Scc*mec*IV occurred, but both were negative for *bbp.* Looking at the antibiotic resistance patterns of the *bbp* positive strains, selective pressure of antibiotics which may affect the frequency of this factor in the bloodstream isolates seems to be unlikely. One of the distinguishing features of CA-MRSA is that high percentage of such strains is positive for PVL (Witte et al. 2005). In our collection of bloodstream isolates, the *PVL* gene occurred occasionally. Moreover, the frequency of this gene in *bbp*-positive subpopulation, versus *bbp*-negative subpopulation, was similar. As a consequence, an association could be excluded.

To sum up, our study shows that Bbp is not strictly associated with BSI. However, from the results one may conclude that for clonally related *S. aureus* strains most commonly causing BSI, the risk of Bbp-mediated complications of septicemia is expected to be higher than for other strains. We found the clonal complex widely distributed among invasive *S. aureus* strains in Europe as to be strongly associated with Bbp. In fact, the high frequency of the *bbp* gene in the most prevalent clone could mean that Bbp contributes to the disease. Although, coincidental presence of this gene in the clone, which is common for other reasons than the carriage, may not be excluded (Aamot et al. 2012).
